# Position of Social Determinants of Health in Urban Man-Made Lakes

**DOI:** 10.5539/gjhs.v5n6p100

**Published:** 2013-09-04

**Authors:** Parisa Shojaei, Masoud Karimlou, Farahnaz Mohammadi, Hosein Malek Afzali, Ameneh Setareh Forouzan

**Affiliations:** 1 Social Determinants of Health Research Center, University of Social Welfare and Rehabilitation Sciences, Tehran, Iran; 2 School of Public Health, Tehran University of Medical Sciences, Tehran, Iran

**Keywords:** man-made lakes, social determinants of health, health impact assessment, content analysis, qualitative study

## Abstract

**Background and Objective:**

A social determinants approach proposes that enhancing living conditions in areas such as income, housing, transportation, employment, education, social support, and health services is central to improving the health of urban populations. Urban development projects can be costly but have health impacts. The benefit derived from the creation of man-made lakes in developing countries is usually associated with great risks; however, the evidence for physical and non-physical health benefits of urban man-made lake is unclear. The aim of this paper is to formulate a conceptual framework of associations between urban man-made lakes and social determinants of health.

**Method:**

This study was a qualitative study carried out using one focus group discussion and 16 individual interviews. Data were analyzed based on deductive-inductive content analysis approach.

**Results:**

Participants’ points of view were analyzed within 261 codes. Data analysis matrix was the conceptual framework of social determinants of health commission and its sub-groups, thus, two structural and mediating determinants categories as well as their sub-sets were created accordingly. In addition, some extra sub-sets including environment, air quality, weather changes, noise pollution, pathogenesis, quality of life, shortage of available resources, region popularity, ethnicity, tourism, social and physical development of children, unintentional injuries, aesthetic, and spirituality were extracted beyond the matrix factors, which were placed in each of above categories based on their thematic content.

**Conclusion:**

This paper has illustrated that the quality and type of man-made lake provided within communities can have a significant and sustained impact on community’s health and wellbeing. Therefore, in order to strengthen positive effects and reduce negative effects of any developmental projects within community, their impacts on public health should be taken into consideration.

## 1. Introduction

In order to enhance public health, health should be taken into account in projects, programs, and policies of others sectors (Improving Health in the United States: The Role of Health Impact Assessment, 2011). Health effects assessment is defined as “combination of methods, procedures, and tools through which can judge about the potential effects of strategies, policies, plans or projects on public health as well as its distribution among the population”. Health effects assessment has come to show that health and welfare are widely influenced by factors outside the health sector itself (Jaine, 2008). During the recent years, many of the countries have reached to an agreement on Health Impact Assessment (HIA) as a tool to hint on the importance of potential effects of health. As a matter of fact, this devise has been introduced in order to operationalize the approach “health in all policy” as well as estimation of results and clarifying the various impacts of a policy on health (Mahoney, Simpson, Harris, Aldrich, & Stewart-Williams, 2011). Urban development programs, alike other projects and plans should be evaluated from health perspective and their effects on public health need to be addressed as well. The relationship between health and its determinants including transportation, social solidarity, housing, and air and water quality with urban planning can help in choosing proper built environment indices as well as monitoring their progress and effectiveness through evaluating the effects of proposed plans, policies, programs, and projects on health (Northridge & Sclar, 2003). Considering health-related objectives in urban planning guarantees its quality through the effects it has on population’s health and life level. Healthy urban planning has come to interfere in enhancing public health and well-being, and has a lot in common with sustainable development principles. Life in a healthy environment means having adequate housing, meaningful and safe life, access to schools, parks, and public spaces, safety and freedom of violence, unpolluted air, soil, and water. Urban planning plays a vital role in formation of environmental, societal, and economical health within the communities (GRAHAM, FILIPPI, & RONSMANS, 1996; Johnson & Freeland, 2011). Reports provided by knowledge network of world health organization (WHO) regarding urbanism stress the importance of structural and mediating social determinates in urban planning. Anecdotes indicate that investment in healthy city can bring back a considerable economic return, which this was also verified by macroeconomics and health commission of WHO in 2001 (Kjellstrom et al., 2007)). A valuable way of understanding the influence of urban spatial factors is to focus on four key factors of urban form and character. These four explain the majority of the range of urban diversity. For each of these, the evidence for health dangers and benefits, existing in urban settings, can be defined as mediated through the spatial planning and urban design. These elements of urban planning include land use pattern, transportation, green space, and urban design (Grant, 2010). In major urban areas around the world, health impact assessment is being used to support and deliver healthy and sustainable communities (Vohra, 2007). Despite a wide range of studies on health effects of urban development projects, a few studies have been carried out on public spaces, which also, according to Scotland country, include artificial lake and its surrounding. They mostly assess green spaces like parks and pay less attention to urban artificial lakes (“Greater Wellington Regional Council – Terms of Reference for the Wellington Regional Strategy “Open Spaces Working Group”, February 2009; Kobelt et al., 2011). Based on what Santa found out in the study namely “assessment of health effects of walk able urban green spaces,” these spaces affect public health both directly and indirectly and is going to have a correlation with health status of local residence as well as environmental quality improvement (Santana, Santos, & Costa, 2009). Results of utilizing HIA in the process of urban planning of Manukau in New Zealand indicated that; it reinforces intersectional cooperation, especially from the structural perspective, among city councils, interested individuals, and out-side agencies, which this procedure is rarely seen in urban planning (Field, 2011). Increasing trend of urbanism in Iran, which points at citizens’ health, is in dire need of collaboration between two efficient knowledge of urban planning and health so that population can live safe within the cities (Khalilabadi, 2006). A few studies have centered on this issue in Iran. Health in development projects of Iran is being looked like a marginal index alike other economic, social, and cultural indices … instead of being in the center of the preparation, approval, and implementation processes of these designs. With this in mind, Iran’s health ministry and medical education, according to its mission for health and equity improvement and with consideration to the act of seventh session of supreme health and food safety council as well as paragraph (b) of article 36 of fifth economic, social and cultural developmental plan, has taken the responsibility of establishment of national health standards for wide developmental projects (Biffi et al., 2011). Therefore, placing health issues in urban projects and plans is essential. Designing and building artificial lake in developing counties usually follows many dangers. Stakeholders need to sit around a table and have active participations in order to utilize the full potential of lake and water sources projects and improve and maintain them. Thus, it necessitates the collaboration of not also engineers but also other health sector experts such as biologists, sociologists, and economists due to important roles of each in planning and implementing developmental projects (Araoye, 2002). Creating the biggest artificial lake in North West of Tehran, in region 22 of municipality, is an instance of these projects under construction. It has three islands along with entertainment, sports, games, and green spaces. These artificial lakes are created with the aim of providing places for tourist absorption, increasing recreational aspects of area, and escalating happiness spirit among population, but they will fail if not placing bioenvironmental and health issues in designing, implementing and utilizing these projects. Considering what mentioned above, a study namely “Health impact assessment of man-made lake in city development strategy on health and social determinants of health” was done, and this article has been taken from it with an intend to determine social determinants of health in the current study.

## 2. Methods and Materials

This qualitative study was designed and carried out with a content analysis approach. Data were collected through individual interviews as well as focus group discussions (FGDs) from September to March 2012 respectively. Individual interviews were planned on 16 informants with following features: educated subjects with at least B.A degree and experts on health and its social determinants features; experts on urban development, environment, water sources, sociology and urban planning, and civil engineering; managers and administrative-executive experts of region 22 of municipality of Tehran; and experts of “Shahran Sazeh” company with at least one-year work experience, lake margins residents, and local authorities (managers of local health houses). Focus group discussions were held with the presence of 10 experts from municipality in housing, transportation, urban planning, civil engineering, environment areas alongside managers of artificial lake with at least one-year work experience. Informants were selected using purposive sampling and data gathering process was continued until data saturation. The focus group discussion was selected because was high ability to explore people’s opinions, concerns, attitudes and experiences of individuals regarding a specific subject matter (Barbour & Kitzinger, 1998; Dahlgren, Emmelin, & Winkvist, 2007). Individual interviews lasted for 50 minutes. Focus group discussion guiding questionnaires and in-depth individual interviews were used to document the data. These procedures were developed based on the research team’s viewpoints and experts who were familiar with urban development and health issues as well as library sources and objectives of the study. These questionnaires dealt with concepts regarding various health dimensions of artificial lake construction. Validity of questionnaires was certified through a pilot study, and required content, sequence, and timing reforms applied to them after this process. We arranged sessions with municipality to make them aware of the study and asked for any permission required. In addition, informants were clarified about the whole research, from its objectives to the methods and confidentiality of the data, and all agreed to participate voluntarily. Main investigator managed the whole interviews. We held FGD in the office of surrogate of mayor with all informants agreed on. We started from general and comprehensive questions in the individual interviews and FGDs, and it progressed to detailed questions by the time. The interviews and FGDs were recorded and written down in the papers word-by-word. We used Graneheim approach for data analysis) Graneheim & Lundman, 2004). The transcribed documents were handled for their meaning units, and were finally coded. Next, codes were extracted and grouped based on the meaning units represented by informants and their similarities and discrepancies, then from these groupings, themes were dug out which represented the main messages carried by the focus group data. Having a list of usual social determinants and main categories, data analysis was managed using content analysis matrix (Averill, 2002). Then, new classes were extracted as analysis continued and they were put in the body of the total data analysis. All codes and themes were checked mutually by research team, and a summary of transcripts was given to the informants in the end. To objectivize the data, simultaneous coding was set out by two researchers, and those codes and classes were compared together. To guarantee trustworthiness of findings, data were documented according to the age, gender, and education of each informant separately. Additionally, amalgamation of data collection and individual interviews as well as FGDs with each other, long-standing relationship with participants and research topic, documenting comprehensive report of research, reviews by participants and observers were other methods for assuring data trustworthiness. Research ethics was observed through receiving written informed consent, getting ethical permission to collect and record the data, maintaining the anonymity of the participants, confidentiality of information and the right to withdraw from the study were considered. And ethical approval from the ethics committee of university. Also, the result of the study was sent to all stakeholders.

## 3. Findings

**Figure 1:**
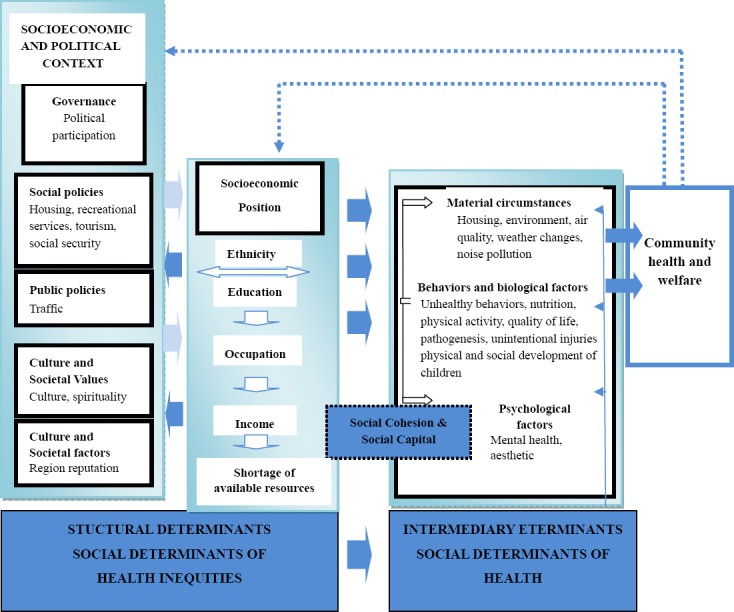
Conceptual framework of social determinants of health in urban man-made lakes

This study was conducted in region 22 of Tehran municipality, west part, in 2012. Average age of participants was 30 years old. They were experts in urban development, environment, water sources, civil engineering, sociology, urban planning, as well as health determinates. Their education varied from B.A to Ph.D., and four of them lived in the studied region and the rest were from other parts of Tehran. Qualitative analysis of data resulted in 261 codes. We considered the structure of conceptual framework of “social determinants of health” commission alongside its sub-sets as data analysis matrix (Solar & Irwin, 2010). Thus, two “structural” and “mediating” groups were achieved, and their sub-groups were determined as well. Therefore, the structural category included Governance, Macroeconomic Policies, Social Policies, Public Policies, Culture, Societal Values, and Socioeconomic Position. In addition, the latter included Material Circumstances, Behaviors, Biological Factors, and Psychosocial Factors. Then, categories taken from codes (sub-categories) were presented under each related category (Figure 1).

Moreover, some other sub-categories including environment, air quality, weather changes, noise pollution, pathogenesis, quality of life, shortage of available resources, region popularity, physical and social development of children, unintentional injuries, aesthetic, and spirituality were extracted as meta-matrix factors, which were placed in either structural or mediating classes based on thematic content. According to findings, sub-category of “political participation” was positioned in governance matrix. This category, in the widest sense, and its process includes definition of needs, forms of discrimination, civil society involvement, and liability/transparence in public administration. Participants believed that any element that provides leisurely and mental relaxation for residents in one way or another could pave the way for social involvement, particularly from political kind. Indeed, they meant that recreational spaces of lake provide opportunities for residents to get together and involve in political negotiations. Toward this issue, one of the experts mentioned, *“public spaces of city in most of the developing countries represent political display.”* Others indicated that whatever strengthens family’s bone, will definitely stylize political spaces. Some others also pointed out that due to existence of special halls and saloons for holding seminars, meetings, celebrations in one of the islands of this lake, it offers a suitable chance for holding political-social meetings in the country.

Sub-strata of housing, recreational services, tourism, and social safety were situated in social policies category. It affects aspects such as labor, social well-being, land, and housing sharing. Findings showed that lake construction affects housing distribution, and is going to have influences on housing and land prices as well. One participant said, *“land and housing selling flourishes because of residential attraction of the area, and the price of housing and land increases due to proximity to lake.”*

Recreational services were another content extracted from data analysis, which we placed it in the “social-political” sub-category. Since, it relates to social welfare of population and is a part of population-based services and facilities; therefore, we put it in sub-category of social-economic determinants (N. H. Committee, 1998). Participants declared that recreational spaces bring welfare for residents if well managed. From municipality’s point of view, one of the goals of constructing lake was to increase ecologic potential through creating and promoting recreational facilities, which reduce commitment of crime by youth through filling their free times.

“Tourism” sub-set, according to its definition, was positioned in recreational services category. This sub-set indicated that artificial lake construction causes the creation of a touristy and recreational place. One participant pointed out, *“learning foreign language is one of the positive effects of tourists for residents through which they can improve their abilities in this regard.”* Furthermore, “social safety” sub-category was situated in “social policies” group. Informants mentioned that gregariousness of people by the lake as a recreational place paves the way for committing crimes and endangers financial and physical safety of people. According to one informant, *“if these spaces are not going to be managed properly, they will become a place for rabbles.”*

We put “traffic” sub-category in “public policies” matrix, because transportation, in definition, sets with public sector. This level also implies other policies in education, health care, and water areas. According to the findings, one of the results of implementation of this project was people’s interest in living or spending their free times in this region, which it results in high traffic load for this area. Informants mentioned that not paying attention to traffic issues and roles of passages while planning for the construction of streets and green spaces around the lake would cause problems for transit. An expert noted, *“traffic in the region, mainly on holidays and weekends, has negative effects such as accidents and injuries to people as well as noise pollution, bewilderment, and mental health problems.”*

“Culture and societal values” matrix involves ethical, religious, political, social, and aesthetic morals (Lahelma, Martikainen, Laaksonen, & Aittomäki, 2004). Thus, “culture and spirituality” sub-category is positioned here. Based on “culture” sub-set, construction of this lake widely changes cultural status of residents accommodating here. As one informant explained, *“mutual relationships transfer culture among residents, and they can better get familiar with different languages and accents.”* Participants pointed out that this can also have negative impacts on culture, and pave the way for local tradition changes as well as fading out the traditional customs.

Considering its definition and since in more spiritual cultures God and Spirituality could be well thought out as a value to the culture (“Some examples of cultural values?”, 2013), therefore, we placed “spirituality” sub-class in this matrix. Furthermore, indigenous Cultural Values of the Native Vegetation of NSW found that the cultural significance contains spiritual, medical, and economic ideals (Schnierer, Faulkner, & Fisher, 2001). This sub-set believes that lakes give a very outstanding landscape to the nature, and help those who want to ponder about the relationship between human beings and nature to rest some time in spiritual peace. People come to believe in omnipotence of God when they look at lakes. Based on an article published by Public Health Advisory Committee of New Zealand, region fame was put in socio-cultural factors (P. H. A. Committee, 2004). Thus, we created a sub-category in structural determinants namely socio-cultural factors and we placed “region fame” in this sub-category.

This sub-category demonstrated that lake construction would increase the popularity of region 22 than its former condition. Experts illuminated that this region is the first region, which has received the environmental management system certificate and is the safest one in Tehran, thus lake construction here increases its acceptability among public and stimulate many people to come and live in this region. Sub-sets of ethnicity, education, job, income, and shortage of available resources were included in socio-economic position matrix.

This matrix embraces both resource-based and prestige-based measures, and is related to both childhood and adult societal class position. The first one discusses material and social resources and properties, including salary, wealth, and educational passes.

Ethnicity indicates that increased migration and ethnicity changes as well as combination of population in the region are of those important issues regarding implementation of developmental projects such as lake building and creation of recreational places. One informant came and said, *“those who have come here because of its silence and being a native area, now are worrying about migration growth and ethnicity changes happening here due to its residential and economic attractions resulted from lake building.”* Indeed, lack of formation of local identity in the marginal areas of lake due to changes in social mixture and temporary residence along with recreational aspects of this region was one of the most important concerns of participants. They described that these issues can increase migration of native residents from this region, especially because of bogging down and drying out the lake or due to traffic and high volume of population in the margins of the lake.

“Education” sub-category also comes to say that formerly this region was weak with regard to educational and training centers whereas creation of this lake motivated investors to build such centers around here. Informants mentioned that it is possible to provide useful educational contents for different groups of people coming to visit this lake through posting pamphlets, consultation, playing movies, posters, and holing classes. As one school manager said, *“programs should be arranged for educating children and youth in this region to not also let them visit the lake but also make them familiar with environmental issues like waste management, traffic and other social problems.”* Some of the participants believed that touristy aspect of this lake brings people with various levels of knowledge here, which it has great effects on region literacy.

“Occupation” sub-set infers that lake and business centers around it will provide hundreds of job opportunities. From some participants’ point of view, these opportunities, themselves, have positive effects on mental health of jobless people, improve spirits of families with unemployed youth, and would have economic encouraging impacts on families and society as a whole. One expert explained, *“these may have negative effects as well and may create some pseudo jobs.”* In addition, an environment expert said, *“some of the jobs provided because of lake construction and its surrounding facilities include aquarium creation, building residential facilities as well as establishment of fish farming pools.”*

Income was another sub-class involved in this matrix. It shows that building lake generates income, which is in result of creating benefits in the region, especially usable aspects of lands around the lake, increased income of business centers working side-by-side the lake, and earning money from tourism industry boom. According to one expert, *“economically, tourism development in region 22, absorbs foreign currency, prevents currency exit, speeds up money flow, enhances public employment, increases per capita income of individuals, and finally it improves quality of life of residents.”*

On the other hand, informants pointed out the negative sides of this phenomenon as it can decline financial income of both recreational centers and tourism industry in west part of Tehran as well as money generated from economic activities and tourism, from “Davoud” shrine to Shahreh Ziba. We also put sub-set of “shortage of available resources” in this matrix. According to participants, building this lake and increased number of population in result, residences and non-residences, as well as high use of public resources such as gas, water, and electricity created problems regarding availability of these assets. One environment expert noted, *“using natural gas for services provided within or around the lake, and considering the required water for artificial lake and its tourists, predicted population of the region and green spaces accordingly, may create some problems.”*

Mediating determinants stratum refers to factors that participate in implementation of structural determinants. This stratum involves material circumstances, behaviors and biological factors, and psycho-social factors. “Housing” sub-category was placed in material circumstances matrix from physical situations, environmental, quality of air, weather changes, and noise pollution perspectives. This matrix comprises determinants related to the physical environment, such as housing (relating to both the lodging itself and its location), consumption potential, i.e. the fiscal means to buy healthy food, warm dress, etc., and the physical working and neighborhood surroundings. Depending on their quality, these circumstances both offer resources for health and hold health hazards as well.

“Housing” sub-stratum suggests that most people believed that as housing price increases, its quality will fall down, especially considering standards in its building as well as its physical conditions. This is because, on one hand, buyers cannot afford these houses, and, on the other hand, housing manufactures should reduce the prices so that they can sell them. To do this, they decide to use cheap and low-quality materials in the buildings.

European Environment Agency (2013) positioned quality of air and water alongside noise pollution in living and working conditions (material circumstances) (*Environment and human health. Joint EEA-JRC report*, 2013), which is a part of mediating determinants. Therefore, these sub-strata belong to this matrix.

“Environment” sub-category infers that changing this region to a touristy place due to building the lake and the plurality of visitors make the role of environment management important. Some of the participants concerned about contamination of environment by wastes produced by passengers and visitors, which this hurts its beauty, such as what happened for North Sea. Also, experts believed that facilities within and around the lake would also increase the amount of generated wastewater, and, in result, it would threaten individuals’ health and sanitation. Meanwhile, if water does not circulate properly, it will leave negative effects. An expert explained, *“ecologic reserves as well as forest and herbal covers of the region (Chitgar park) will be reduced because of the increased volume of traffic, visitors, and pollution and problems created in the result of their presence.”*

The most important and positive effect of lake, according to participants, was its role in filtration of the air. They stated that lake, through making humidity in the region, cleans and cool the air, which it ultimately has significant impacts on people’s health. An environment expert declared, *“Creation of artificial lakes in metropolitan areas absorbs tourists bedsides increasing the green space per capita and cleaning the air.”* Weather changes sub-set also demonstrated that lake is effective on this phenomenon as well. According to informants, lake manufacture helps save a high volume of water in the region, which it absorbs sun heat, facilitates thermal exchange with the environment, transfers temperature to the deepest and lowest layers of the water surface, reflects sun rays, and reacts against evaporation. In environment expert’s point of view, *“we can save natural ecosystem through knowing the mutual effects of lake and environment and their changes, and utilizing them in improving the air quality and natural environment as well as decreasing the range of temperature variations in the region.”*

Regarding “noise pollution” sub-set, although tourists and visitors have positive effects on the region, recreational aspect of this place brings in a high range of population on holidays, which their noise and crowd influence individuals’ mental health and cause them insomnias, particularly at night. Some participants, in contrast, believed that these noises, especially created by children playing around, have promising mental and psychological effects on the residents beside the lake.

Sub-categories of unhealthy behaviors, nutrition, and physical activities, known as lifestyle (N. H. Committee, 1998), along with quality of life and pathogenesis, unintentional injuries as well as physical and social development of children were placed in “behaviors and biological factor” matrix.

This contains smoking, diet, alcohol use, and absence of physical workout, which again can be either health keeping and enhancing (such as exercise) or health injuring (cigarette smoking and fatness); among biological factors we are involving genetics factors, as well as from the angle of social determinants of health, age, and sex distribution. “Lifestyle” sub-stratum shows that people’s lifestyle becomes better because of exercise, and making planned decisions can either facilitate or endanger their lifestyle, predominantly among women.

Participants declared their concerns about emergence of some unhealthy and anti-cultural behaviors, mostly among young boys and girls because of specific environment and forestry spaces around the lake. Regarding nutrition sub-category, informants mentioned that there are possibilities to sell fast foods to youth in this place, but their food safety can be saved through selling natural and nutritious foods. Also, some informants hinted on the importance of educational programs for healthy nutrition advertisement. Findings indicated that construction of lake and provision of a proper environment for sports, water games, and walking as well as bicycle lane around the lake increase physical and mental activities of people and decrease their physical problems, particularly mentally. One participant mentioned, *“Increased physical and social interactions improves physical and mental health, especially among housewives, and reduce osteoporosis among them.”*

Moreover, “quality of life” also demonstrated that this feature of people living permanently near the lake was decreased in this region in result of increased traffic, pollution, and population density. From some others’ point of view, tourism development in region 22 absorbs foreign currency, prevents currency exit, speeds up money flow, enhances public employment, increases per capita income of individuals, and finally it improves quality of life of residents.

“Pathogenesis” represents prevalence of specific diseases with water origin in result of lake construction. One informant said, *“Anyway, since lake is a natural ecosystem, it is possible that some diseases emerge through its water or poor hygiene of visitors, and tourists can also bring in some contagious diseases with themselves.”* Participants believed that water-source diseases in the region and prevention methods should be handled before lake construction. Furthermore, unintentional injuries imply that these incidences are natural in such places. Participants expressed that some of these injuries happen because of accidents resulted from high traffic in the region, and some others are related to physical injuries happen for children while playing. In one informant’s point of view, *“there are dangers of sinking into the lake for residents, park visitors, mainly children and youth.”*

We situated “physical and social development of children” sub-set in mediating determinants category (Organization, 2005). This sub-set is going to tell us that children, because of their social interactions with their friends, grow better in such places, and they develop mentally. An expert expressed, *“here provides a good chance for education of various social issues such as environmental, and traffic matters, cultural subjects like cities and different countries traditions etc. to children in order to help them learn together beside recreation.”* “Mental health” and “aesthetic” sub-categories were included in psychological factors matrix. This comprises psychosocial stress makers (for instance, adverse life events and job pressure), stressful living situations (e.g. high debt), and absence of social backing, managing styles, etc. Mental health was one of the very important issues mentioned by informants. They expressed that water brings tranquility for human beings, and recreational spaces along with pleasant bowers influence people’s psyche, make them happier, and improve their psychological diseases. To one informant, *“doing health recreations and interesting aquatic games as well as social interactions among people help them free their mind from problems and bring happiness to them.”* Some of the informants also believed that the sounds of children’s excitement and joy have definite effects on residents’ mind. Aesthetic is a potential for better understanding of things, and it changes individuals’ attitudes and makes them think optimistically, therefore, it better sets with “mental health” matrix. This sub-category implies that this lake can play an effective role in development, renovation, and beautification of the surrounding environment. According to experts, other impacts of lake construction include positive mental sound effects it has on residents and visitors because of its beautiful landscapes, islands as well as its huge fountains. “Social capital” placed itself between structural and mediating determinates. It says that such open and public spaces provide chances for families and friends to get together and reach emotional safety in this way. Some pointed out that this place could increase social capital and give people the chance of togetherness, which it ultimately results in improved mental health of people. One participant mentioned, *“Families, especially housewives and elderlies come together and this is good for them.”*

## 4. Discussion and Conclusion

Results of present study indicated that, based on the conceptual framework of social determinants of health commission, lake construction affected structural and mediating elements as well as their sub-categories. Moreover, sub-classes of “environment”, “quality of air”, “weather variations”, “noise pollution”, “pathogenesis”, “quality of life”, “shortage of available resources”, “popularity of the region”, “ethnicity”, “tourism”, “social and physical development of children”, “unintentional injuries”, “aesthetic”, and “spirituality” were extracted as factors beyond the matrix, and were included in each structural or mediating elements based on their concepts.

Similar studies have also maneuvered on these elements. South Carolina Institute of Medicine and Public Health (IMPH) evaluated the health effects of park, forest paths, and green spaces plans in west part of Greenville, and found out physical activities; social capital and solidarity; economic stability of families and society; housing; access to food; public and individual safety; and quality of air and water as important determinants associated with these spaces (South Carolina Institute of Medicine and Public Health (IMPH), 2013). Furthermore, Greater Wellington Regional Public Health has mentioned four social, environmental, economic, and cultural categories in its assessment of open spaces effects on health, and concluded that these four determine cause- effect pathways between open spaces with health and welfare. For instance, in social area, this included increased physical activity and social cohesion, encouraging to play and enjoy, in economic sector, high economic development, increased land values as well as job opportunities, in environmental area, supporting biodiversity, environmental resources management, and in cultural domain, it involved cultural identity, increased access to nature (Greater Wellington Regional Council, 2009). Faculty of Public Health of London reported enhancement of welfare and mental health for all, increased physical activity, reduced violence, and anti-social behaviors, decreased health disparities, declined mortality and cardiovascular diseases, improvement of air quality, solving noise pollution, and fiscal profits as health determinants associated with green spaces (Faculty of Public Health, 2010). Lee and Maheswaran (2011), in their study namely “the health benefits of urban green spaces”, concluded that there exists weak evidences to support the relationship between physical and mental health with urban green spaces, whereas access to and quality of green spaces affect physical activities of people, and in fact, the age, gender, ethnicity, and safety perception of people are important in this regard, too. In addition, Morris (2003) expressed that some of the environmental benefits of natural open and green spaces include filtering air pollution, stabilization of ground surfaces, the interception of rainfall, which decreases flooding, the creation of visual and sound obstacles, the provision of temporary cover for run-down sites, encouraging the sustainability of wildlife habitats, emotional well-being, reduced stress, improved health, increased physical activity and quality of life, enhanced personal and social communication skills, improved spiritual, sensory, and aesthetic awareness, and ability to assert personal control and increased sensitivity to one’s own well-being. Green space Scotland (2008) found out that presence of green spaces significantly affects tourism, biodiversity, social solidarity, management of environmental resources, learning and education, community safety and development, land value, capital and fiscal development, recreation and enjoyment, physical activity, mental health as well as community health elements. Increased activity was seen as a common feature of our study with other studies, and it almost is the common findings of all researches done on making changes in fabricated environment with an aim to provide more access to recreational areas such as creation of parks and green spaces. Measures of the built environment that are connected with physical activity comprise existence of bicycle and pedestrian infrastructure, parks, street network density, residential density, land use mix, and urban plan (Saelens & Handy, 2008; Saelens, Sallis, & Frank, 2003; Sallis et al., 2009). Of common findings of Greater Wellington Regional Public Health, green space Scotland, and South Carolina Institute of Medicine and Public Health (IMPH) was increased solidarity and social capital. Nowadays, it is believed that success or failure of urban planning in creating and changing public spaces is assessed through counting the number of women and men using these facilities as well as various characteristics of people using such spaces. If these assessments result in satisfying consequences, that time we would claim that these spaces could play an important role in increasing and improvement of social interactions and reduction of deprivations caused by social, ethnical, age, and sex strata (Gaderi, 2005). According to Kawachi et al. (1997), public open spaces are perfect settings to promote social unity and inclusion and thus for enhancing ‘social capital’, a factor strongly connected with good health. *“The aspect of social capital that makes it a classic public good is its property of non-excludability; that is, its benefits are available to all living within a particular community, and access to it cannot be restricted”*. As mentioned earlier, tourism influences fiscal stability and income generation in the region. Greater Wellington Regional Public Health, green space Scotland, and South Carolina Institute of Medicine and Public Health (IMPH) as well as London public health faculty have come to an agreement on financial benefits of tourism. A study carried out by a professor from Texas A&M University demonstrated that out of 25 studies investigating the role of parks in property values, 20 showed a growth of property values near a park or open space. How much the property value increases depends on numerous factors, containing the size and type of open space and its distance from open space (Reed, 2012). Considering this potential increase in assets values, it is important to offer several housing choices that range in price to accommodate all who live in the society. Providing affordable housing choices is vital to communities, and these choices should meet the needs of all residents regardless of their age or income level (Environmental Impacts Analysis Unit Minnesota Department of Health, 2012). According to the findings, increased housing price in result of lake construction makes problems for vulnerable families living in this region. This finding was supported by Greater Wellington Regional Public Health, green space Scotland, and South Carolina Institute of Medicine and Public Health (IMPH), and they pointed out that a growth in assets values that a park can offer to a community could pave the way for the possibility of displacement due to gentrification. This shift can have a significant influence on health differences, especially for the poor, women, and children, the elderly and racial minorities. Studies show that these vulnerable groups have proclivities for higher rates of asthma, diabetes, and cardiovascular disease. Those residents who are influenced by such shift can experience an alteration in stress levels, crime, and/or mental health. Other health related impacts of this displacement comprise lack of access to healthy food choices, transport, and quality of schools, bicycle, and walking pathways as well as affordable housing (South Carolina Institute of Medicine and Public Health (IMPH), 2013). As mentioned by green space Scotland and IMPH, public and green spaces, such as lake, strongly affect people’s safety. Brien et al. argued that sense of safety is a recurring theme in many studies examining the relationship between green space and health, particularly in urban settings. There is some evidence that urban forests and green spaces more generally contribute positively to sense of safety (O’Brien, Williams, & Stewart, 2010). Our findings found cleaning the air and pollution reduction as two main roles of man-made lake. Based on green space Scotland, South Carolina Institute of Medicine and Public Health, Faculty of Public Health, and Morris, an initial effect of any place having clean water and green space is reducing air pollution and cooling the weather. Urban designers and planners are aware of the strong effects of aquatic bodies, even small and narrow like lake, on quality of life in cities, because they include internal and intrinsic aesthetic aspects, and have recreational and educational values, which light urban area up (Zhang, Liu, & Yang, 2005). In his article namely “protection and management of urban lakes”, Rao (2009) argued that these lakes affect economic, recreational, and aesthetic values of region, and make the air cool and clean. Other common result of current study and studies of Morris, Faculty of Public Health, and green space Scotland, is increase of mental health. Round found that positive neighborhood settings are associated with positive determinants of mental health, such as parks for walking, while negative neighborhood settings are associated with negative determinants of mental health, such as stressors from chronic contact with motor vehicle traffic and noise (Round, 2013). According to the findings, one of the necessities of lake construction in district 22 of Tehran was its recreational tourism aspect. This was also supported by green space Scotland. Since urban tourism requires planning, management to guarantee its positive effects, and reduction of adverse consequences, therefore, integrated and comprehensive planning as well as effective managerial strategies are needed to reach these goals. Urban tourism should be managed trough integrated and appropriate planning-based methods. Hall and Pitch see the effects of tourism on physical environment of cities as follows:

*“Destruction of lands because of tourism development, urban hydrological changes, visual effects, development of tourism areas, creation of new architectural methods, and reinforcement of local architectural forms* (Bakhshi, 2011). Using lakes and their surrounded green spaces require observation of bioenvironmental issues as well as waste management. This helps reduction of risks, water-source diseases, and tourism related problems. People’s right for free access to water pathways and aquatic areas help them meet their needs, and this is considered as their right in some countries, whereas this freedom in using resources can destroy the environment, too (Maller et al., 2008). Sorensen (1996) in a study on lakes of Concepción in Chile mentioned that bioenvironmental issues of lakes could be a serious and problematic issue. There are seven lakes in this city. Entrance of sewage and increased nutrients within the lakes, predominantly small lakes, causes problems for urban texture of other neighboring areas. Destroying water quality, stench, stagnant water, insect overpopulation, bothering mice, and increased aquatic weed growth are some of these problems. Thus, lakes in this city not also did not increase recreation and enjoyment, but they also destroyed environmental conditions. What’s more, only the way that it was planned created residential pattern, which destroyed lake landscape. Moreover, illegal residential places were built around these lakes and they ruined the quality of water.

From a social perspective, the most important consideration about lakes should begin with their stakeholders (people who spend their free times by the lakes, those who play role in building and management of man-made lakes, those who use water of these lakes). According to Nakagami (1991), difficulties of proper lake management come from its stakeholders. All organizations and stakeholders should be aware of merits and demerits of constructing lakes and management of their water, and all agreements and commitments should be arranged in advance between stakeholders (Minnesota Lakes Association, 2000). Nowadays, urban management has become aware of social, cultural, environmental, and mental affairs of citizens instead of paying pure attention to physical issues of cities. Healthier citizens play naturally more role in sustainable development, and vice versa, mental and physical disabilities of citizens are vital barriers in the way of any urban development (Barati, 2011).

## References

[ref1] Araoye P (2002). Man-made lakes, ecological studies and conservation needs in Nigeria. Revista de biología tropical.

[ref2] Averill J. B (2002). Matrix analysis as a complementary analytic strategy in qualitative inquiry. Qualitative Health Research.

[ref3] Bakhshi H (2011). Tahlile asarbakhshie modiriate shahri dar tose gardeshgarie kalanshahre Tehran. Thesis submitted in fulfilment of the Ph.D of Geographic & Urban Planning.

[ref4] Barati N (2011). Roykardhaye jadid dar barnamerizi, modiriate shahri: Barnamereizie shahre salem.

[ref5] Barbour R, Kitzinger J (1998). Developing focus group research: politics, theory and practice.

[ref6] Biffi M, Ziacchi M, Bertini M, Gardini B, Mazzotti A, Massaro G, Corsini D (2011). How to truly value implantable cardioverter-defibrillators technology: Up-front cost or daily cost?. International Journal of Technology Assessment in Health Care.

[ref7] Center for Urban Regional Affairs (2000). Minnesota Lakes Association. Sustainable lakes planning workbook: a lake management model.

[ref8] Committee N. H (1998). The social, cultural and economic determinants of health in New Zealand: action to improve health.

[ref9] Committee P. H. A (2004). A guide to health impact assessment: a policy tool for New Zealand.

[ref10] Dahlgren L, Emmelin M, Winkvist A (2007). Qualitative methodology for international public health.

[ref11] Environmental Impacts Analysis Unit Minnesota Department of Health Environmental Health Division (2012). http://www.health.state.mn.us/divs/hia/docs/slp_hia.pdf.

[ref12] Environmental Impacts Analysis Unit Minnesota Department of Health Environmental Health Division (2011). http://www.health.state.mn.us/divs/hia/docs/slp_hia.pdf.

[ref13] Faculty of Public Health (2010). Great Outdoors: How Our Natural Health Service Uses Green Space To Improve Wellbeing.

[ref14] Field A (2011). Integrating Health Impact Assessment in Urban Design and Planning: The Manukau Experience.

[ref15] Gaderi S (2005). Barresie payamadha va natayeje barnamehaye pishgiri az jorm dar fazahaye omomie shahri ba takid bar “tarhe ertegae amniate ejtemaei”.

[ref16] GRAHAM W. J, FILIPPI V. G. A, RONSMANS C (1996). Demonstrating programme impact on maternal mortality. Health Policy and Planning.

[ref17] Graneheim U. H, Lundman B (2004). Qualitative content analysis in nursing research: concepts, procedures and measures to achieve trustworthiness. Nurse education today.

[ref18] Grant M, Barton H, Coghill N, Bird C (2010). Evidence Review, Spatial Determinants of Health in Urban Settings. Paper presented at the Building Health, Planning and designing for health and happiness conference.

[ref19] Greater Wellington Regional Council (2009). Terms of Reference for the Wellington Regional Strategy “Open Spaces Working Group”.

[ref20] Jaine R (2008). Health Impact Assessment of the Regional Policy Statement: regional form and energy draft provisions.

[ref21] Johnson A, Freeland P (2011). Growth Management Planning in a Low Growth Environment—Dunedin’s Spatial Plan. Paper presented at the NZPI Conference - Winds of Change.

[ref22] Kawachi I, Kennedy B. P, Lochner K, Prothrow-Stith D (1997). Social capital, income inequality, and mortality. American journal of public health.

[ref23] Kjellstrom T, Mercado S, Sattherthwaite D, Mccgranahan G, Friel S, Havemann K (2007). Our cities, our health, our future: Acting on social determinants for health equity in urban settings. Our cities, our health, our future: Acting on social determinants for health equity in urban settings.

[ref24] Kobelt G, Lekander I, Lang A, Raffeiner B, Botsios C, Geborek P (2011). Cost-effectiveness of etanercept treatment in early active rheumatoid arthritis followed by dose adjustment. International Journal of Technology Assessment in Health Care.

[ref25] Lahelma E, Martikainen P, Laaksonen M, Aittomäki A (2004). Pathways between socioeconomic determinants of health. Journal of Epidemiology and Community Health.

[ref26] Lee A, Maheswaran R (2011). The health benefits of urban green spaces: a review of the evidence. Journal of public health.

[ref27] Mahoney M, Simpson S, Harris E, Aldrich R, Stewart-Williams J (2011). Equity-focused health impact assessment framework.

[ref28] Maller C, Townsend M, Henderson-Watson C, Pryor A, Prosser L, Moore M (2008). Healthy Parks Healthy People: The health benefits of contact with nature in a park context.

[ref29] Morris N (2003). Health, well-being and open space. Literature Review. OPEN space: the research centre for inclusive access to outdoor environments.

[ref30] Nakagami K.-I (1991). Interest Groups Involved with the use of Water Resources/Environments.

[ref31] National Research Council of the National Academies (NRCNA) (2011). Improving Health in the United States: The Role of Health Impact Assessment.

[ref32] Northridge M. E, Sclar E (2003). A joint urban planning and public health framework: contributions to health impact assessment. American Journal of Public Health.

[ref33] O’Brien L, Williams K, Stewart A (2010). Urban health and health inequalities and the role of urban forestry in Britain: A review: The Research Agency of Forestry commission.

[ref34] Organization W. H (2005). Towards a conceptual framework for analysis and action on social determinants of health. Secretariat Commission on Social. Determinants of Health.

[ref35] Publications Office of the European Union (2013). Environment and human health. Joint EEA-JRC report.

[ref36] Rao R. R (2009). Conservation & Management of Urban lakes (water bodies): Concerns and Strategies. http://en.wikipedia.org/wiki/Lakes_in_Bangalore.

[ref37] Reed J (2012). Greenville Hospital System Swamp Rabbit Trail: Year 1 Findings. http://www.upstateforever.org/newsviews_other/SRTImpactStudyYear1.pdf.

[ref38] Round M (2013). Health Impact Assessment Of The Massachusetts Department Of Transportation (MassDOT) Grounding McGRATH Study Draft Release For Public Comment.

[ref39] Saelens B. E, Handy S. L (2008). Built environment correlates of walking: a review. Medicine and science in sports and exercise.

[ref40] Saelens B. E, Sallis J. F, Frank L. D (2003). Environmental correlates of walking and cycling: findings from the transportation, urban design, and planning literatures. Annals of behavioral medicine.

[ref41] Sallis J. F, Saelens B. E, Frank L. D, Conway T. L, Slymen D. J, Cain K. L, Kerr J (2009). Neighborhood built environment and income: examining multiple health outcomes. Social science & medicine (1982).

[ref42] Santana P, Santos R, Costa C (2009). Walkable Urban Green Spaces: Health Impact Assessment in Amadora, Portugal’. REAL CORP 2009: CITIES 3.0–Smart, Sustainable, Integrative Strategies, Concepts and Technologies for Planning the Urban Future.

[ref43] Schnierer S, Faulkner A, Fisher C (2001). ‘Aboriginal Cultural Values of the Native Vegetation of New South Wales: A background paper of the Native Vegetation Advisory Council of New South Wales: Department of Land and Water Conservation, Background Paper 5, NSW.

[ref44] Scotland G (2008). Health Impact Assessment of Greenspace, A Guide. Greenspace Scotland. www.greenspacescotland.org.uk/upload/File/Greenspace%20HIA.pdf.

[ref45] Solar O, Irwin A (2010). A conceptual framework for action on the social determinants of health.

[ref46] Some examples of cultural values? (2013). http://wiki.answers.com/Q/Some_examples_of_cultural_values.

[ref47] Sorensen H. A (1996). Managing urban lakes: An integrating experience. International Journal of Water Resources Development.

[ref48] South Carolina Institute of Medicine and Public Health (IMPH) (2013). A Health Impact Assessment (HIA) of Park, Trail, and Green Space Planning in the West Side of Greenville, South Carolina.

[ref49] Vohra S (2007). International perspective on health impact assessment in urban settings. New South Wales Public Health Bulletin.

[ref50] Zhang F.-L, Liu J.-L, Yang Z.-F (2005). Ecosystem health assessment of urban rivers and lakes for six lakes in Beijing. Acta Ecologica Sinica.

